# Pathogens detected from patients with acute respiratory infections negative for SARS-CoV-2, Saitama, Japan, 2020

**DOI:** 10.5365/wpsar.2023.14.4.1057

**Published:** 2023-12-15

**Authors:** Kodai Miyashita, Hayato Ehara, Kyoko Tomioka, Kazue Uchida, Hirokazu Fukushima, Tsuyoshi Kishimoto, Asao Honda

**Affiliations:** a Saitama Institute of Public Health, Saitama, Japan.

## Abstract

**Objective:**

During the coronavirus disease pandemic in Japan, all patients with respiratory symptoms were initially tested for severe acute respiratory syndrome coronavirus 2 (SARS-CoV-2). This study describes the respiratory pathogens detected from patients who tested negative for SARS-CoV-2 at the Saitama Institute of Public Health from January to December 2020.

**Methods:**

We performed pathogen retrieval using multiplex real-time polymerase chain reaction on samples from patients with acute respiratory diseases who tested negative for SARS-CoV-2 in Saitama in 2020 and analysed the results by age and symptoms.

**Results:**

There were 1530 patients aged 0–104 years (1727 samples), with 14 pathogens detected from 213 patients (245 samples). Most pathogens were human metapneumovirus (25.4%, 54 cases), rhinovirus (16.4%, 35 cases) and *Mycoplasma pneumoniae* (13.1%, 23 cases). Human metapneumovirus, human coronavirus (but not NL63) and *M. pneumoniae* were detected in almost all age groups without any significant bias. Seasonal human coronaviruses, human metapneumovirus, *M. pneumoniae* and several other pathogens were detected until April 2020.

**Discussion:**

Multiple respiratory pathogens were circulating during 2020 in Saitama, including SARS-CoV-2 and influenza viruses. We suggest introducing a system that can comprehensively monitor the regional prevalence of all viruses that cause acute respiratory infections.

In December 2019, severe acute respiratory syndrome coronavirus 2 (SARS-CoV-2) was first identified in a cluster of pneumonia cases in Wuhan, China, (*1*) with the illness later termed coronavirus disease (COVID-19). The number of cases rapidly increased worldwide, and there were repeated waves of the epidemic. (*2*, *3*) The first case in Japan was diagnosed on 15 January 2020. (*4*) In Saitama Prefecture, the first case was reported on 1 February 2020.

Respiratory viral infections mainly follow a seasonal pattern, with an annual increase and cessation of the epidemic in response to changes in temperature and humidity. However, the prevalence of seasonal respiratory viral infections significantly decreased during the COVID-19 pandemic, although the infections did not completely disappear. (*5*-*8*) Factors that caused this decrease in Japan included the implementation of personal protective measures – such as wearing masks, encouraging handwashing and avoiding crowds and confined spaces – and the change in attitudes of patients towards receiving medical care and the responses of medical institutions. (*9*-*11*) Unlike other countries, Japan did not mandate lockdowns of the population; instead, residents were encouraged to cooperate with the recommended countermeasures.

Saitama Prefecture is part of the Kanto region in eastern Japan. It is located north of Tokyo, covering an area of 3797 km^2^. As of 1 January 2020, its population was 7 344 765, of whom 858 384 were aged < 15 years and 1 934 994 were aged ≥ 65 years. (*12*)

In Japan, during the initial period of the COVID-19 pandemic, the clinical priority for patients with respiratory symptoms or fever was to test for SARS-CoV-2 to ensure patients received appropriate care and to prevent further transmission. Therefore, little is known about pathogens other than SARS-CoV-2 that caused respiratory tract infections during this period. In this study, we report on the detection of various respiratory pathogens in samples from patients who tested negative for SARS-CoV-2 at the Saitama Institute of Public Health from January to December 2020.

## Methods

### Sample selection

Samples sent to the Saitama Institute of Public Health from 30 January to 31 December 2020 that tested negative for SARS-CoV-2 were included in the study. These comprised nasal, pharyngeal and nasopharyngeal swabs; nasal discharge; tracheal aspirate; alveolar lavage fluid; and sputum from people suspected to have COVID-19. As suspected influenza cases are usually confirmed via antigen testing at the clinical site and only positive samples are sent to public health reference laboratories, such specimens were assumed to contain influenza viruses and were excluded. (*13*)

The cases’ symptoms and age and the date of sample collection were recorded on the laboratory forms collected with the samples. Samples from cases among children aged < 15 years were included if they had at least one symptom of fever, upper respiratory tract infection or lower respiratory tract infection (LRTI) reported on the laboratory form; samples from cases aged ≥ 15 years were included if they had at least one symptom of LRTI reported on the laboratory form.

The number of pathogens detected was tabulated by sample collection date. Cases were divided into three age groups for evaluation, namely paediatric (< 15 years), intermediate (≥ 15 years to < 65 years), and elderly people (≥ 65 years), and the presence of LRTI was assessed in each group.

### Pathogen detection procedures

RNA was extracted from specimens using an automated nucleic acid extraction system (EZ1 Advanced XL; QIAGEN, Venlo, Netherlands). Influenza A and B viruses, rhinovirus, adenovirus, enterovirus, human *Parechovirus*, human metapneumovirus, seasonal human coronaviruses (OC43, 229E, HKU1 and NL63), parainfluenza virus types 1–4, human respiratory syncytial virus (RSV), human bocavirus and *Mycoplasma pneumoniae* were detected using a multiplex real-time reverse transcription–polymerase chain reaction (rRT–PCR) kit (FTD Respiratory Pathogens 21 assay; Siemens Health care, Erlangen, Germany). If the samples were positive for influenza virus or RSV, the type or lineage was determined by rRT–PCR. If samples were positive for adenovirus, enterovirus or human *Parechovirus*, genotyping was performed using Sanger sequencing.

## Results

### Detected pathogens

**Fig. 1:**
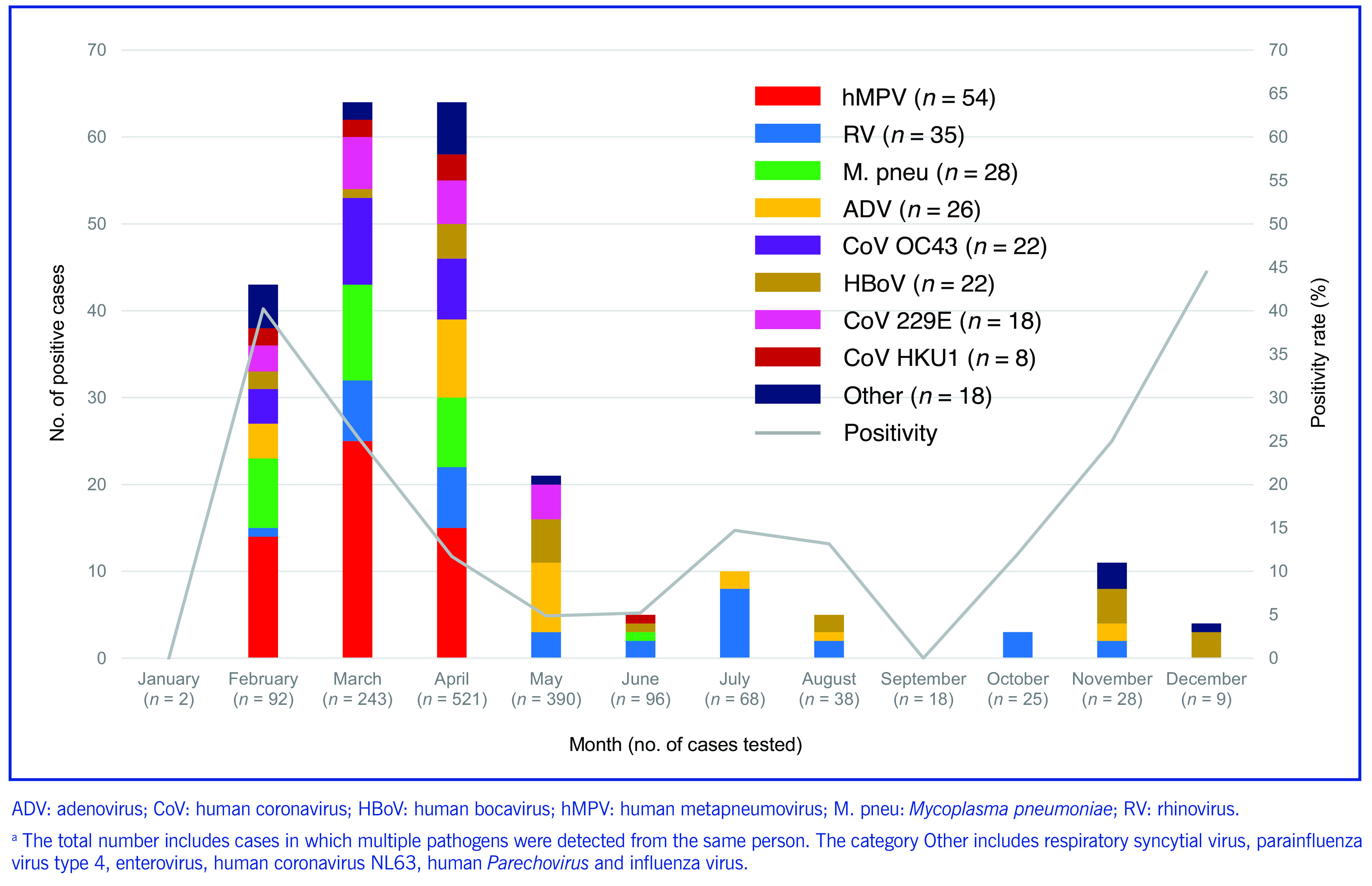
Number of individual respiratory pathogens detected and positivity rate in samples that tested negative for SARS-CoV-2, by month, Saitama, Japan, 2020^a^

There were 1727 samples from 1530 cases tested during the study period. From these, 14 different pathogens were detected in 245 samples from 213 cases (13.9% of all eligible cases) (**Fig. 1**). Human metapneumovirus was the most frequently detected pathogen, detected in 67 samples from 54 cases (25.4% of 213 positive cases). Rhinovirus and *M. pneumoniae* were detected in, respectively, 38 samples from 35 cases (16.4% of 213) and 34 samples from 28 cases (13.1% of 213). These three pathogens accounted for more than half of the detected pathogens (54.9%, 117 cases). Seasonal human coronaviruses were detected in 58 samples from 50 cases (23.4% of 213 positive cases), and included OC43 detected in 24 samples from 22 cases (10.3%), 229E detected in 21 samples from 18 cases (8.4%), HKU1 detected in 11 samples from 8 cases (3.7%) and NL63 in 2 samples from 2 cases (0.9%).

[**insert** Figure 1]

### Seasonal differences

Testing was most frequently performed between February and May, with 81.4% of cases (1246/1530) tested during this period (**Fig. 1**). The highest positivity rate was observed in December (44.4%, 4/9 cases), followed by February (40.2%, 37/92 cases), March (25.5%, 62/243 cases) and November (25.0%, 7/28 cases). Human metapneumovirus, RSV, seasonal human coronaviruses and *M. pneumoniae* were detected most frequently between February and May (**Fig. 1**).

### Detection of multiple pathogens

Two different pathogens were detected in 16 cases and three different pathogens were detected in one case (**Table 1**).

**Table 1:** Cases with multiple respiratory pathogens detected in samples that tested negative for SARS-CoV-2, Saitama, Japan, 2020

Case no.	Respiratory pathogens detected and type	Patient age group (years)	LRTI symptoms	Collection month
1	Human bocavirus Human *Parechovirus*, nt Adenovirus, nt	0–9	–	November
2	Human bocavirus Coxsackievirus A4	0–9	–	May
3	Human bocavirus Coronavirus 229E	0–9	+	November
4	Human bocavirus Adenovirus type 1	0–9	+	May
5	Human bocavirus Human *Parechovirus*, nt	0–9	–	November
6	Human bocavirus Human metapneumovirus	0–9	+	March
7	Human bocavirus Rhinovirus	0–9	+	April
8	Adenovirus type 3 Human metapneumovirus	0–9	+	April
9	Adenovirus, nt Respiratory syncytial virus type B	0–9	–	March
10	Adenovirus type 3 Influenza virus B Victoria	0–9	–	April
11	Coronavirus OC43 *Mycoplasma pneumoniae*	0–9	+	March
12	Adenovirus, nt *Mycoplasma pneumoniae*	0–9	+	February
13	Coronavirus HKU1 Parainfluenza virus type 4	20–29	+	February
14	Coronavirus OC43 *Mycoplasma pneumoniae*	30–39	+	February
15	Adenovirus, nt Human metapneumovirus	40–49	+	February
16	Coronavirus OC43 Human metapneumovirus	40–49	+	March
17	Coronavirus 229E Respiratory syncytial virus type B	80–89	+	March

LRTI: lower respiratory tract infection; nt: not typed.

### Virus typing results

Adenoviruses were detected in 26 cases (12.1% of 213 positive cases). These included adenovirus type 1 (7 cases), followed by adenovirus type 2 (3 cases), adenovirus types 3 and 4 (2 cases each) and adenovirus type 6 (1 case); 11 cases could not be typed.

RSV was detected in seven cases (3.3% of 213): RSV-A in four cases (1.9%), RSV-B in two cases (0.9%), and one case could not be typed. Parainfluenza viruses were detected in four cases (1.9%), all type 4. Enterovirus was detected in two cases (0.9%), and coxsackievirus group A type 4 and coxsackievirus group B type 3 were detected in one case each (0.5% each). Human *Parechovirus* was detected in two cases (0.9%), both of which could not be typed. Influenza virus (B/Victoria lineage) was detected in one case (0.5%).

### Detection results by age group

Patients’ ages ranged from 0 to 104 years, with a median age of 69 years (interquartile range, 39–82 years); 904 patients were male (59.1%), 618 were female (40.4%) and the sex of eight patients was unknown (0.5%). The highest number of samples tested was from patients aged 80–89 years (22.5%, 343/1530), although the positivity rate was only 8.7% (30/343 cases) (**Table 2**).

**Table 2:** Number of cases, number of samples and positivity rate for respiratory pathogens among cases that tested negative for SARS-CoV-2, by age group, Saitama, Japan, 2020

Patient age group (years)	No. of cases	No. of samples	Proportion of total cases (%)	No. of positive cases	Positivity rate (%)
0–9	190	192	12.4	77	40.5
10–19	54	55	3.5	11	20.4
20–29	52	62	3.4	9	17.3
30–39	96	116	6.3	18	18.8
40–49	110	132	7.2	23	20.9
50–59	113	136	7.4	10	8.8
60–69	162	185	10.6	14	8.6
70–79	294	339	19.2	14	4.8
80–89	343	389	22.5	30	8.7
≥ 90	116	121	7.6	7	6.0
Total	1530	1727	100	213	13.9

Children aged 0–9 years had the highest positivity rate, with pathogens detected in 40.5% (77/190) of cases. This was followed by those aged 40–49 years (20.9%, 23/110 cases), 10–19 years (20.4%, 11/54 cases) and 30–39 years (18.8%, 18/96 cases).

Among those aged 0–9 years, the most frequently detected pathogens were rhinovirus (68.5%, 24/35 cases), adenovirus (65.4%, 17/26 cases), human bocavirus (95.5%, 21/22 cases) and RSV (42.9%, 3/7 cases), and enterovirus and human *Parechovirus* (2 cases each) and influenza B virus (1 case) were detected only in this age group.

*M. pneumoniae* was most frequently detected among those aged 30–39 years (32.1%, 9/28 cases), and human metapneumovirus was most frequently detected among those aged 40–49 years (24.0%, 13/54 cases). Seasonal human coronaviruses (OC43, 229E, HKU1 and NL63) were most frequently detected among those aged 80–89 years (32.0%, 16/50 cases). Parainfluenza virus was most frequently detected among those aged ≥ 90 years (50.0%, 2/4 cases) (**Table 3**).

**Table 3:** Number of positive cases and number of samples of respiratory pathogens from cases that tested negative for SARS-CoV-2, by age group and pathogen, Saitama, Japan, 2020

Patient age group (years)	No. of positive cases (no. of samples) by pathogen														
	hMPV	RV	ADV	CoV OC43	HBoV	CoV 229E	CoV HKU1	RSV	PIV4	EV	HPeV	CoV NL63	Influenza virus	*M.* *pneumoniae*	Negative
0–9	9 (9)	24 (24)	17 (18)	1 (1)	21 (21)	3 (3)	1 (1)	3 (3)	1 (1)	2 (2)	2 (2)	1 (1)	1 (1)	4 (5)	113 (113)
10–19	1 (2)	2 (2)	0	0	0	0	0	0	0	0	0	0	0	8 (8)	43 (43)
20–29	3 (4)	1 (1)	1 (1)	1 (1)	0	1 (1)	1 (2)	0	1 (1)	0	0	0	0	1 (2)	43 (50)
30–39	3 (4)	2 (3)	0	2 (3)	0	1 (1)	1 (1)	0	0	0	0	1 (1)	0	9 (12)	78 (92)
40–49	13 (17)	1 (1)	1 (1)	3 (3)	0	2 (3)	1 (2)	0	0	0	0	0	0	4 (5)	87 (102)
50–59	7 (8)	0	1 (1)	0	0	1 (2)	0	0	0	0	0	0	0	1 (1)	103 (124)
60–69	5 (7)	1 (1)	1 (1)	4 (4)	0	2 (2)	0	1 (2)	0	0	0	0	0	0	148 (168)
70–79	2 (3)	3 (5)	3 (3)	2 (2)	0	0	2 (3)	1 (1)	0	0	0	0	0	1 (1)	281 (321)
80–89	9 (10)	1 (1)	2 (2)	8 (9)	1 (1)	6 (7)	2 (2)	2 (2)	0	0	0	0	0	0	313 (356)
≥ 90	2 (3)	0	0	1 (1)	0	2 (2)	0	0	2 (2)	0	0	0	0	0	108 (113)
Total^a^	54 (67)	35 (38)	26 (27)	22 (24)	22 (22)	18 (21)	8 (11)	7 (8)	4 (4)	2 (2)	2 (2)	2 (2)	1 (1)	28 (34)	1317 (1 482)

ADV: adenovirus; CoV: human coronavirus; EV: enterovirus; HBoV: human bocavirus; hMPV: human metapneumovirus; HPeV: human *Parechovirus; M. pneumoniae: Mycoplasma pneumoniae;* PIV4: parainfluenza virus type 4; RSV: respiratory syncytial virus; RV: rhinovirus.

^a^ Columns do not add up to the total as multiple pathogens were detected in some cases and samples.

### Classification by age group and symptoms

Based on classifications by age group and the presence of LRTI, the positivity rate observed in the paediatric group with LRTI was 52.0% (39/75 cases); that in the paediatric group without LRTI was 28.6% (46/161 cases); that in patients with LRTI in the intermediate group was 15.9% (69/433 cases) and that in elderly people was 6.9% (59/861 cases).

Human metapneumovirus and three seasonal human coronaviruses (OC43, HKU1 and NL63) were detected only in patients with LRTI, whereas rhinovirus, adenovirus and human bocavirus were more frequently detected in patients without LRTI (**Table 4**). *M. pneumoniae* was more common in children with LRTI and in the intermediate age group. Although a degree of difference was observed in the positivity rate between the elderly and intermediate age groups, there was no marked difference in the pathogens detected, except *M. pneumoniae*.

**Table 4:** Number of cases, number of samples and positivity rate for respiratory pathogens among people who tested negative for SARS-CoV-2, by age group and presence of lower respiratory tract infection, Saitama, Japan, 2020

Age group	LRTI symptoms	No. of cases tested (no. of samples)	No. of positive cases (no. of samples)	Positivity rate (%)	No. of positive cases (no. of positive samples) by pathogen														
					hMPV	RV	ADV	CoV OC43	HBoV	CoV 229E	CoV HKU1	RSV	PIV	EV	HPeV	CoV NL63	Influenza virus type B	*M.* *pneumoniae*	Negative
0–14	+	75 (77)	39 (41)	52.0	9 (9)	9 (9)	6 (7)	1 (1)	7 (7)	2 (2)	1 (1)	2 (2)	0	0	0	1 (1)	0	8 (9)	36 (36)
	−	161 (161)	46 (46)	28.6	0	17 (17)	11 (11)	0	14 (14)	1 (1)	0	1 (1)	1 (1)	2 (2)	2 (2)	0	1 (1)	2 (2)	115 (115)
Total^a^	-	236 (238)	85 (87)	36.0	9 (9)	26 (26)	17 (18)	1 (1)	21 (21)	3 (3)	1 (1)	3 (3)	1 (1)	2 (2)	2 (2)	1 (1)	1 (1)	10 (11)	151 (151)
15–64	+	433 (513)	69 (89)	15.9	28 (36)	4 (5)	4 (4)	9 (10)	0	5 (7)	3 (5)	1 (2)	1 (1)	0	0	1 (1)	0	17 (22)	364 (424)
≥ 65	+	861 (976)	59 (69)	6.9	17 (22)	5 (7)	5 (5)	12 (13)	1 (1)	11 (11)	4 (5)	3 (3)	2 (2)	0	0	0	0	1 (1)	802 (907)
Total^a^	-	1294 (1 489)	128 (158)	9.9	45 (58)	9 (12)	9 (9)	21 (23)	1 (1)	15 (18)	7 (10)	4 (5)	3 (3)	0	0	1 (1)	0	18 (24)	1166 (1 331)

ADV: adenovirus; CoV: human coronavirus; EV: enterovirus; HBoV: human bocavirus; hMPV: human metapneumovirus; HPeV: human *Parechovirus*; LRTI: lower respiratory tract infection; *M. pneumoniae: Mycoplasma pneumoniae*; PIV: parainfluenza virus; RSV: respiratory syncytial virus; RV: rhinovirus.

^a^ Totals do not include multiple pathogens detected from the same case and sample.

## Discussion

We detected a variety of pathogens in samples from patients who had acute respiratory symptoms but had tested negative for SARS-CoV-2 in 2020 in Saitama, Japan. Public health and social measures implemented to prevent SARS-CoV-2 transmission might have changed the circulation of seasonal infectious diseases in various regions, (*5*-*8*) and the COVID-19 pandemic itself might have suppressed the spread of other respiratory viruses. (*14*)

The detection of non-SARS-CoV-2 respiratory pathogens in children suggests that other viruses – such as rhinovirus, adenovirus and human bocavirus – should also be considered in the differential diagnosis of upper respiratory tract infections in children. Differences in viral stability between non-enveloped and enveloped viruses, such as seasonal human coronaviruses and human metapneumovirus, may affect differences in detection. (*15*) Additionally, non-enveloped viruses have been detected in paediatric patients and are believed to circulate in immunologically susceptible age groups, raising concerns about outbreaks in the future when nonmedical interventions, such as mask-wearing, are lifted. (*5*-*8*) Seasonal human coronaviruses have been reported as being more prevalent during winter and early spring; (*16*) however, in this study, they were not detected during winter in the second half of 2020.

Although weekly reports of the viruses isolated and the detection of cases of upper and lower respiratory inflammation in Japan indicated that respiratory infections spread throughout 2019, (*17*, *18*) the decrease in the number of pathogens detected after June 2020 can be partly attributed to the decline in samples received at the public health laboratory. The Ministry of Health, Labour and Welfare issued a notice on 2 June 2020 allowing PCR testing of saliva samples for SARS-CoV-2, (*19*) after which the number of respiratory tract samples sent to our laboratory drastically decreased.

During the study period, testing for SARS-CoV-2 was limited and controlled by legislation or institute-specific rules. (*20*, *21*) In addition, when a patient suspected of having COVID-19 tested negative for SARS-CoV-2, the need for further pathogen testing was determined by the examining doctor. Not knowing about the circulation of respiratory pathogens other than SARS-CoV-2 during this period is problematic for respiratory pathogen surveillance in Japan. (*11*, *22*)

By testing patients with suspected COVID-19 for other viruses that cause acute respiratory infections, we have provided a summary of infections caused by other viruses with similar symptoms. Critical surveillance gaps may be filled by having a more systematic process through which public research institutions such as ours can test samples from cases with influenza-like illness and acute respiratory infections to provide information about prevalence, contagiousness and severity of the disease. (*23*) We propose there is a need to introduce a system that can comprehensively monitor the regional prevalence of all viruses that cause acute respiratory infections, and we hope that the results of this study will be used as a resource to improve surveillance.
